# Supplementary material for: Dynalign II: common secondary structure prediction for RNA homologs with domain insertions
Source: Nucleic Acids Res. 2014 Nov 21;42(22):13939–48. doi: 10.1093/nar/gku1172 (PMC4267632; doi:10.1093/nar/gku1172)
Supplement: SUPPLEMENTARY DATA [file supp_gku1172_nar-02021-z-2014-File012.zip › manual/RNA_class/html/class_turbo_fold-members.html]

RNAstructure Classes: Member List

- Main Page
- Classes

- Class List
- Class Hierarchy
- Class Members

# TurboFold Member List

This is the complete list of members for TurboFold, including all inherited members.

|  |  |  |
| --- | --- | --- |
| fold(double gamma=0.3, int n\_iterations=3, int \_n\_parallel\_pfunctions=1) | TurboFold |  |
| GetErrorCode() | TurboFold |  |
| GetErrorMessage(int err\_code) | TurboFold |  |
| GetErrorString(int err\_code) | TurboFold |  |
| GetNumberSequences() | TurboFold |  |
| GetPair(const int i\_seq, const int i, const int structurenumber=1) | TurboFold |  |
| GetPairProbability(const int i\_seq, const int i, const int j) | TurboFold |  |
| MaximizeExpectedAccuracy(const int i\_seq, const double maxPercent, const int maxStructures, const int window, const double gamma=1.0) | TurboFold |  |
| PredictProbablePairs(const int i\_seq, const float probability) | TurboFold |  |
| ProbKnot(const int i\_seq, const int n\_iterations, const int minhelixlength) | TurboFold |  |
| ReadSHAPE(const int i\_seq, const char fp[], const double par1, const double par2) | TurboFold |  |
| SetMaxPairingDistance(int distance) | TurboFold |  |
| SetProgress(TProgressDialog &Progress) | TurboFold |  |
| SetTemperature(double temp) | TurboFold |  |
| StopProgress() | TurboFold |  |
| TurboFold(const char fasta\_fp[]) | TurboFold |  |
| TurboFold(vector< string > \*sequences, vector< string > \*saves) | TurboFold |  |
| WriteCt(const int i\_seq, const char fp[]) | TurboFold |  |
| ~TurboFold() | TurboFold |  |

---

Generated on Wed Sep 7 12:53:49 2011 for RNAstructure Classes by 
 1.5.7.1
